# Säugling mit beidseitiger Hornhauttrübung und Aniridie

**DOI:** 10.1007/s00347-020-01051-3

**Published:** 2020-02-07

**Authors:** Helena Wagner, Uta Matysiak, Wolf A. Lagrèze

**Affiliations:** 1 Klinik für Augenheilkunde, Universitätsklinikum Freiburg, Killianstr. 5, 79106 Freiburg, Deutschland; 2 Medizinische Fakultät, Albert-Ludwigs-Universität Freiburg, Freiburg, Deutschland; 3 Klinik für Allgemeine Kinder- und Jugendmedizin, Universitätsklinikum Freiburg, Freiburg, Deutschland

## Fallbericht

### Anamnese

Ein 10 Wochen alter weiblicher Säugling wurde in unserer Ambulanz vorgestellt. Den Eltern des Mädchens waren sofort nach Geburt beidseits weite Pupillen bei fehlender Iris und ein weißer zentraler Fleck auf beiden Hornhäuten aufgefallen. Es bestand zu dem Zeitpunkt kein Nystagmus. Systemische Erkrankungen waren nicht bekannt. Eine sonographische Untersuchung der Bauchorgane ergab einen Normalbefund.

**Figure 1:**
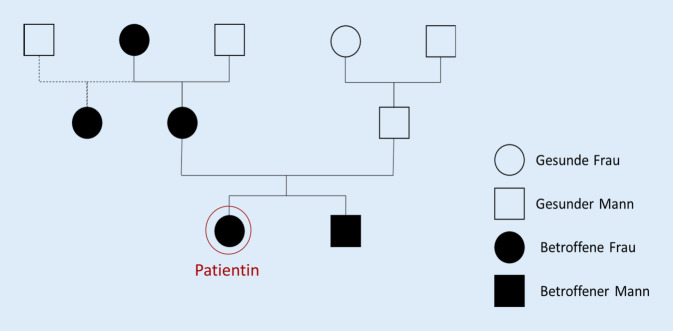

Die Familienanamnese ergab, dass sowohl der Bruder der Indexpatientin als auch weitere Angehörige mütterlicherseits einen ähnlichen Phänotyp zeigten, jedoch ohne Hornhauttrübung (Abb. 1). Die Mutter hatte einen Visus von 0,2 beidseits. Die Großmutter mütterlicherseits hatte zudem ein Glaukom. Systemische Erkrankungen waren auch in der Familie nicht bekannt.

### Klinischer Befund

**Figure 2:**
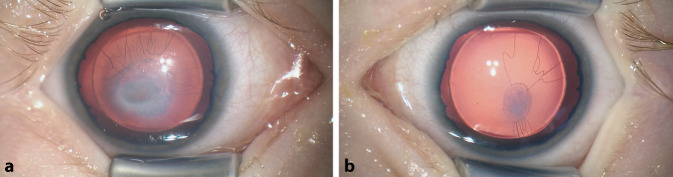

Aufgrund des jungen Alters führten wir eine Untersuchung in Allgemeinanästhesie durch. Unter dem Operationsmikroskop zeigte sich eine geringe, periphere korneale Vaskularisation ähnlich einer Limbusstammzellinsuffizienz. Beidseits fehlte die Iris. Es bestand eine zentrale, links stärker als rechts ausgeprägte Adhäsion von Linse und Hornhaut, die mit einer Trübung der Hornhaut einherging. Im peripheren Teil waren Hornhaut und Linsen beidseits klar. Zirkulär zogen von schmalen Irisresten feine Gefäße zu den Trübungen (Abb. 2). In der Fundoskopie zeigten sich an beiden Augen eine Makulahypoplasie und eine mäßige Optikushypoplasie links mehr als rechts. Der Durchmesser der Hornhaut betrug beidseits 10,5 mm. Eine automatische Messung der Bulbuslänge war nicht möglich, sonographisch konnte sie auf 17 mm beidseits geschätzt werden. Der Augendruck lag bei Messung mittels Tonopen bei 22 mm Hg beidseits.

## Wie lautet Ihre Diagnose?

Aufgrund der Symptomassoziation von beidseitigem Fehlen der Iris, zentraler Hornhauttrübung und korneolentikulären Adhäsionen stellten wir die Diagnose einer beidseitigen Aniridie bei Peters-Anomalie Typ II.

### Definition

#### Aniridie

Aniridie ist eine schwerwiegende Fehlbildung, welche alle Augenabschnitte betrifft. Sie tritt mit einer Prävalenz von 1:64.000 bis 1:96.000 auf [2]. Pathognomonisch ist das Fehlen oder die variable Hypoplasie der Iris. Eine foveale Hypoplasie sowie eine Optikushypoplasie mit reduzierter Sehschärfe sind häufig vorhanden und in der Regel mit einem sensorischen Defektnystagmus verbunden. Das Ausmaß der Makulahypoplasie beeinflusst dabei den Visus und das Ausmaß des Nystagmus. Weitere Zeichen sind eine Keratopathie aufgrund einer Limbusstammzellinsuffizienz, eine Katarakt und das Glaukom. Aniridiepatienten können andere sensorische Defizite entwickeln. Am häufigsten zeigt sich ein reduzierter Geruchssinn, ebenso können Hörstörungen auftreten [2]. Wenn die Aniridie durch eine Deletion des *PAX6*-Gens (inklusive des *WT1*-Gens) verursacht wird, beträgt das Risiko für den Wilms-Tumor, einen kindlichen Nierentumor, 50 % (WAGR-Syndrom). Darüber hinaus sind hierbei multiple, urogenitale Fehlbildungen sowie starke geistige Retardierung charakteristisch.

#### Peters-Anomalie

Die Peters-Anomalie tritt in 60–80 % der Fälle bilateral auf. Ihre Prävalenz liegt bei 1:110.000. Es wird die Peters-Anomalie Typ I, die sich durch eine zentrale Trübung der Hornhaut mit iridokornealer Adhäsion auszeichnet, von der Peters-Anomalie Typ II mit korneolentikulären Adhäsionen unterschieden. Das Peters-plus-Syndrom umfasst die Peters-Anomalie in Verbindung mit einer Mund-Kiefer-Gaumen-Spalte, einer kleinen Statur, abnormen Ohren und geistiger Behinderung.

Der Phänotyp ist sehr variabel: Das Spektrum reicht von einseitigen Fällen ohne assoziierte systemische Fehlbildungen bis zu bilateralen Fällen mit okulären Assoziationen wie Myopie, Irishypoplasie, Glaukom, Mikroophthalmie, Katarakt oder Optikushypoplasie und zahlreichen assoziierten systemischen Fehlbildungen.

#### Kombination von Aniridie und Peters-Anomalie

Sowohl die Aniridie als auch die Peters-Anomalie gehören zur Gruppe der Vorderabschnittsdysgenesien. Nach Nischal lassen sich diese in iridotrabekuläre und keratoiridolentikuläre Dysgenesien einteilen [3]. Beide Arten der Dysgenesie sind auf eine gestörte Neuralleistendifferenzierung zurückzuführen. Die Aniridie grenzt sich hier allerdings von den anderen Fehlbildungen ab, da ihr Ursprung meist in Störungen des Oberflächenektoderms und des Neuroektoderms zu sehen ist. *PAX6 *übt einen indirekten Einfluss auf die Entwicklung der Neuralleiste aus und kann somit auch eine kongenitale Hornhauttrübung verursachen [2]. Die Kombination von Aniridie und Peters-Anomalie als Folge einer *PAX6*-Mutation spricht also für eine phänotypische Variation innerhalb eines Genotyps.

Die in unserem Fall zur Hornhauttrübung hinführenden Gefäße könnten entweder auf die persistierende Tunica vasculosa lentis oder die sekundäre Vaskularisation der Hornhauttrübung zurückzuführen sein. Die Koinzidenz von Aniridie und einer persistierenden Pupillarmembran wurde in einem Fallbericht vorbeschrieben [1].

#### Molekulargenetik

Die Kombination einer Aniridie und einer Peters-Anomalie ist ein sehr seltener Phänotyp. Wichtig ist eine genetische Testung auf die infrage kommenden Gene wie *PAX6, WT1, FOXE3* sowie in seltenen Fällen des *CYP1B1* oder des *PITX2*. Die Mehrheit der Fälle von Aniridie wird durch Mutationen im *PAX6*-Gen verursacht. Das *PAX6*-Gen (engl. „paired box 6 gene“) kodiert einen Transkriptionsfaktor mit mehreren Funktionen in der Entwicklung des Auges und anderer Gewebe. Zusammen mit *PAX2* ist *PAX6* für die Entwicklung der Linse, des Pigmentepithels, der Netzhaut und des Sehnervs verantwortlich. Familiäre Aniridiefälle zeigen eine autosomal-dominante Vererbung mit hoher Penetranz, aber erheblicher phänotypischer Variabilität.

Das *PAX6*-Gen liegt auf dem kurzen Arm des Chromosoms 11 (11p13) in unmittelbarer Nähe des *WT1*-Gens; Mikrodeletionen auf diesem Chromosomenabschnitt, bei denen beide Gene (*PAX6 *und* WT1*) deletiert sind, führen zum WAGR-Syndrom, einem „contiguous gene deletion syndrom“. Diese Möglichkeit muss bei Patienten mit Aniridie in Betracht gezogen werden. Besteht der Verdacht auf ein WAGR-Syndrom oder kann eine *WT1*-Deletion nachgewiesen werden, sind regelmäßige sonographische Kontrollen der Niere notwendig.

### Weiteres Vorgehen

Die Hornhäute und Linsen der kleinen Patientin waren peripher ausreichend klar, sodass von einer Operation abgesehen wurde. Die Erfolgsquote der Keratoplastik bei Peters-Anomalie Typ 2 liegt bei unter 30 %, wobei es Hinweise darauf gibt, dass die Quote bei Typ I höher ausfällt [4]. Es müssen zudem die hohe Abstoßungsrate bei Kindern und die Gefahr des postoperativen Glaukoms bedacht werden. Ferner sind die Makula- und Optikushypoplasie visuslimitierend. Als wichtigste Maßnahmen wurden eine gute Druckkontrolle und regelmäßige Befundkontrollen angesehen. Ferner wurde eine visuelle Frühförderung empfohlen.

**Diagnose:** Aniridie und Peters-Anomalie

Aufgrund des familiär gehäuften Phänotyps der Aniridie wurde primär das dominant vererbte *PAX6*-Gen getestet. Die molekulargenetische Diagnostik zeigte eine heterozygote Spleiß-Mutation im Gen *PAX6* NM_000280.3:c.916+2T>C. Die Variante (rs1131691549) wurde bereits als pathogen beschrieben [5]. Eine *WT1*-Deletion konnte nicht nachgewiesen werden. Bei positivem Nachweis wurde auf die Testung weiterer mit Peters-Anomalie verbundener Gene verzichtet.

## Fazit für die Praxis

- Dysgenesien des vorderen Segments, die durch *PAX6*-Mutation verursacht werden, können phänotypisch sehr variabel sein.
- Bei komplexen Symptomassoziationen sollte neben der genetischen Testung des *PAX6*-Gens auch das *WT1*-Gen untersucht werden zum Ausschluss eines WAGR-Syndroms.
- Bei sporadischem Auftreten der Peters-Anomalie sollte neben einer *PAX6*- und *WT1*-Testung auch eine genetische Testung auf *FOXE3* sowie *CYP1B1* und *PITX2* erfolgen.
